# Frontiers and hotspots of adipose tissue and NAFLD: a bibliometric analysis from 2002 to 2022

**DOI:** 10.3389/fphys.2023.1278952

**Published:** 2023-12-21

**Authors:** Shuxiao Gu, Yanfang Qiao, Susu Liu, Shuangjie Yang, Shibo Cong, Sili Wang, Deshuai Yu, Wei Wang, Xinlou Chai

**Affiliations:** ^1^ School of Traditional Chinese Medicine, Beijing University of Chinese Medicine, Beijing, China; ^2^ Dongzhimen Hospital, Beijing University of Chinese Medicine, Beijing, China

**Keywords:** non-alcoholic fatty liver disease, adipose tissue, bibliometrics, hotspots, CiteSpace, VOSviewer

## Abstract

**Background:** The annual incidence of non-alcoholic fatty liver disease (NAFLD) continues to rise steadily. In recent years, adipose tissue (AT) has gained recognition as a pivotal contributor to the pathogenesis of NAFLD. Employing bibliometric analysis, we examined literature concerning AT and NAFLD.

**Methods:** Relevant literature on AT in NAFLD from 1980 to 2022 was extracted from the Web of Science Core Collection. These records were visualized using CiteSpace and VOSviewer regarding publications, countries/regions, institutions, authors, journals, references, and keywords.

**Results:** Since 2002, a total of 3,330 papers have been included, exhibiting an annual surge in publications. Notably, the quality of publications is superior in the USA and Europe. Kenneth Cusi stands out as the author with the highest number of publications and H-index. *Hepatology* is the journal boasting the highest citation and H-index. The University of California System holds the highest centrality among institutions. References specifically delve into physiological processes associated with AT in NAFLD. Currently, lipid metabolism and inflammation constitute the principal research mechanisms in the AT-based regulation of NAFLD, with pertinent keywords including microRNA, T cell, hypoxia, sarcopenia, hepatokine, gut microbiota, and autophagy. The Mediterranean diet is among the most widely recommended dietary approaches for potential NAFLD treatment.

**Conclusion:** This paper represents the inaugural bibliometric study on the effects of AT on NAFLD, offering valuable insights and directions for future research.

## 1 Introduction

Non-alcoholic fatty liver disease (NAFLD) is clinically defined as a metabolic disease characterized by >5% hepatic steatosis (Kleiner et al., 2005). The current global prevalence of NAFLD is estimated to be approximately 25%, with an estimated 314 million patients with NAFLD expected by 2030 (Estes et al., 2018; Powell et al., 2021). In addition to advancing to liver fibrosis, cirrhosis, and hepatocellular carcinoma, NAFLD elevates the risk of type 2 diabetes (T2DM), cardiovascular disease (CVD), and chronic kidney disease (CKD) (Byrne and Targher, 2015). The pathogenesis of NAFLD is not fully understood, and no specific drugs are currently available for its treatment. Addressing the prevention and therapy of NAFLD has become an urgent concern.

Excess visceral fat, insulin resistance (IR), chronic low-grade inflammation, and alterations in the intestinal flora are reported as pathological manifestations in patients with NAFLD, involving structural and functional changes in adipose tissue (AT), the liver, muscle tissue, and the intestine (van der Poorten et al., 2008). Studies in the last decade have identified that intervening in AT could be an effective treatment for NAFLD, which is regulated through multiple pathways (Sakane et al., 2021). For instance, AT secretes various adipokines, including adiponectin, leptin, resistin, tumor necrosis factor-alpha (TNF-α), and interleukin-6 (IL-6). Leptin possesses anti-adipogenic properties and is inversely correlated with the severity of hepatic steatosis (Musso et al., 2005). Leptin exhibits insulin-sensitizing and anti-steatogenic effects; however, leptin resistance develops with the worsening of the disease, leading to increased inflammation and fibrosis in the liver (Polyzos et al., 2015). TNF-α promotes the release of free fatty acid (FFA) through lipolysis, exacerbating the progression of NAFLD (Cai et al., 2005).

With an increasing number of researchers showing interest in the mechanisms of AT in NAFLD, a large volume of relevant literature has been published. However, systematically capturing the latest research hotspots and trends in the field becomes challenging due to the lack of quantitative and qualitative analyses (Ma et al., 2021). Bibliometrics, an information visualization analysis, employs literature as its research object. Common tools for this purpose include CiteSpace and VOSviewer (Ge et al., 2022). The method performs collaborative network and citation analysis on countries/regions, institutions, journals, and authors in the literature and conducts co-occurrence and cluster analyses of references and keywords (Lv et al., 2023). This approach aids in uncovering the current research status, development process, trends, and frontier dynamics in this field, gaining high credibility in academic research (Ellegaard and Wallin, 2015; Peng et al., 2022). Therefore, this study provides a bibliometric analysis of AT and NAFLD from 2002 to 2022 to provide new perspectives for the future management of NAFLD through the regulation of AT.

## 2 Methods

### 2.1 Data collection

The present study utilized the Web of Science Core Collection (WoSCC) database. The search formula employed was as follows: (TS = (“adipose tissue” OR “fat* tissue*”) OR “lipid tissue*” OR WAT OR iWAT OR gWAT OR SAT OR VAT OR PAT OR BAT OR “beige fat” OR “beige fat” OR BeAT OR “white adipose tissue” OR “brown adipose tissue”) AND TS = (“non-alcoholic fatty liver” OR “non-alcoholic fatty liver” OR “non-alcoholic steatohepatitis” OR “non-alcoholic fatty liver” OR “metabolism-related fatty liver” OR NAFLD OR NASH OR MAFLD)).

We conducted a comprehensive literature search from 1980 to 2022, with an initial screening conducted on 11 April 2023, resulting in the collection of 3,645 records. Among these records, three types of literature were selected: article (*n* = 2,454), accounting for 67.33% of the total records, review article (*n* = 933), and proceeding paper (*n* = 57), totaling 3,444 records. Each record underwent a secondary screening based on titles and keywords, resulting in 3,435 records being obtained. On 12 April 2023, all records were exported and saved as plain text files with the “Full Record and Cited References” format on the WoSCC database webpage. This format includes the full list of information allowed to be downloaded from the webpage.

According to the citation report, there are a total of 94,906 citing articles, with a total of 179,708 citations, an average of 51.57 citations per item, and an H-index of 185. Total citations and H-index are indicators of the contribution of publications in the scholarly community. They can reflect the impact and quality of publications (Grech and Rizk, 2018). After removing duplicates (105 articles) using CiteSpace, a total of 3,330 articles were included in the study.

### 2.2 Data analysis

The main software applications used in this study were CiteSpace 6.2.R1, VOSviewer 1.6.19, SCImago Graphica Beta 1.0.34, Gephi 0.10.1, and Microsoft Office Excel 2019 for subsequent data processing (Chen, 2004; van Eck and Waltman, 2010).

In this study, we analyzed annual and cumulative publications, co-authorship of countries, regions, institutions, and authors, co-cited authors, citations of journals, co-occurrence of references and keywords, and co-citations of references. Co-cited authors refer to two or more authors who were cited by the same subsequent papers. Reference co-citation means that two or more papers were cited by one or more subsequent papers at the same time, indicating a co-citation relationship.

## 3 Results

### 3.1 Analysis of annual/cumulative publications and citations

**FIGURE 1:**
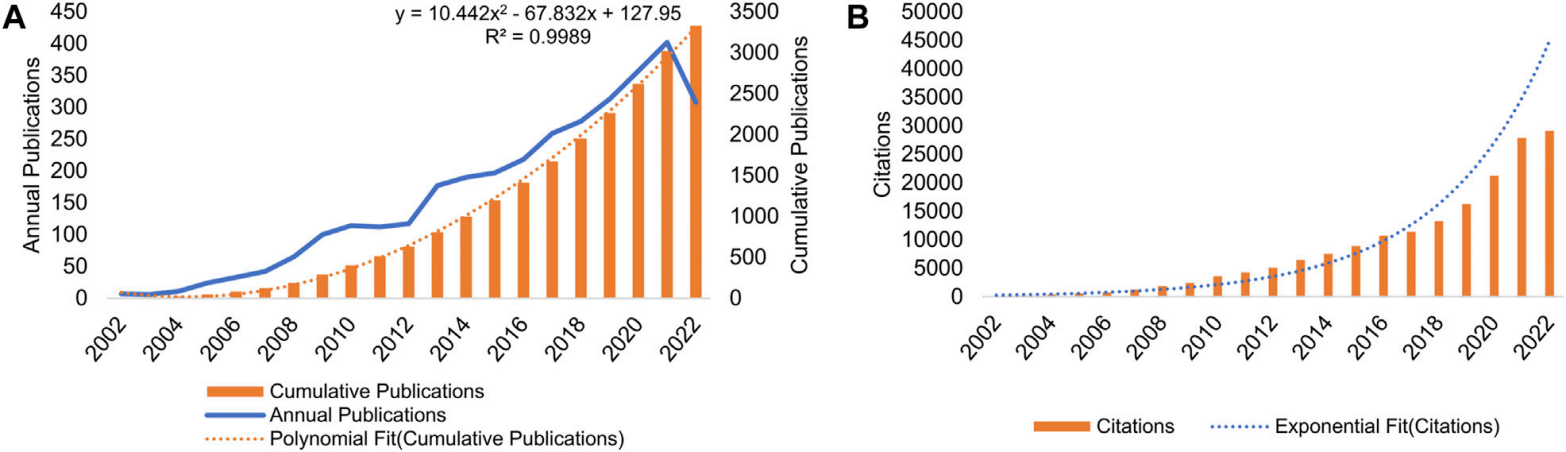
Analysis of the number of publications and frequency of citations in AT and NAFLD. **(A)** Annual/cumulative volume analysis from 2002 to 2022. Trend lines reflect annual growth rates. **(B)** Annual citations analysis from 2002 to 2022.

Figure 1A shows the annual/cumulative volume of publications on AT and NAFLD from 2002 to 2022. The highest number of articles, 402 in total, was published in 2021. Annual publications have been consistently increasing over the years, with a notable surge since 2008. There was a sharp increase in publications between 2012 and 2013. Citations have shown an upward trend from 2002 to 2021 (Figure 1B), and the frequency of citations is increasing, indicating that the field is attracting more and more attention. The R^2^ value of 0.9989 for the polynomial fit of cumulative publications indicates a well-fitted dataset. Using the formula y = 10.442x^2^ − 67.832x + 127.95, the prediction suggests that cumulative publications in AT and NAFLD could reach 5,423 in 5 years and 8,060 in 10 years.

### 3.2 Analysis of countries/regions

**FIGURE 2:**
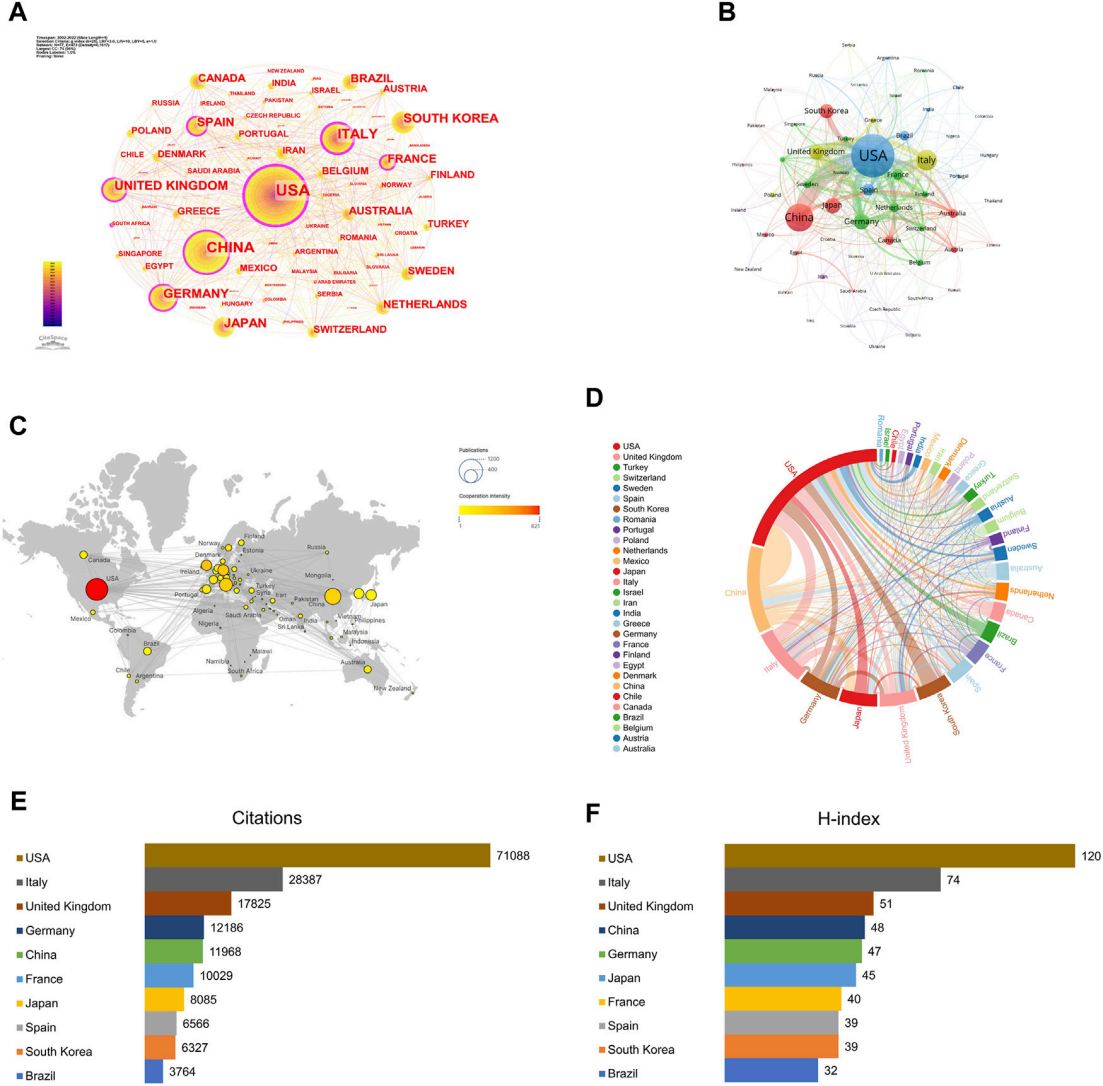
Contribution of studies on AT in NAFLD in different countries/regions. **(A)** Visual map of the cooperative network based on CiteSpace. BC is represented in the diagram as the purple outer circle of the node, which is a measure of the importance of a node. **(B)** Visual map of the cooperative network based on VOSviewer. **(C)** Geographical distribution map of country/region collaboration. **(D)** Chord diagram of country/region collaboration. **(E)** Top 10 countries/regions in terms of citations. **(F)** Top 10 countries/regions in terms of H-index.

CiteSpace was used to visualize the country/region cooperation network, and the parameters were set as follows: time slicing (2002–2022), years per slice (1), node types (country), Top N (50), and selection criteria (k = 25). A network graph with 77 Nodes and 473 links was obtained through CiteSpace (Figure 2A). The top three countries in terms of publications (Table 1) were the USA with 970 (29.13%), China with 520 (15.62%), and Italy with 339 (10.18%). Together, these three countries accounted for 54.92% of the total number of publications, surpassing half of the total, reflecting that mainly researchers from these countries have a stronger interest in AT and NAFLD and more academic results. With a centrality of 0.54, the USA significantly outpaces other countries, holding a position of leadership in the field.

**TABLE 1:** Top 10 countries in terms of publications in AT and NAFLD. The centrality is the measure of influence of nodes in the network, with values greater than 0.1 considered key nodes for analysis.

Rank	Country	Record count	% of 3330	Average article citation	Citation	H-index	Total link strength	Centrality
1	USA	970	29.13	77.63	71,088	120	621	0.54
2	China	520	15.62	20.34	11,968	48	207	0.14
3	Italy	339	10.18	72.87	28,387	74	236	0.21
4	Germany	235	7.06	42.28	12,186	47	250	0.12
5	Japan	223	6.70	34.88	8,085	45	65	0.06
6	United Kingdom	218	6.55	79.98	17,825	51	273	0.14
7	South Korea	202	6.07	31.47	6,327	39	65	0.01
8	Spain	150	4.50	36.02	6,566	39	121	0.11
9	France	137	4.11	69.37	10,029	40	127	0.14
10	Brazil	120	3.60	30.60	3,764	32	39	0.01

The betweenness centrality (BC) of the USA, Italy, France, Germany, Spain, the United Kingdom, and China is relatively high (Figure 2A). The average article citations for the United Kingdom (79.98), the USA (77.63), Italy (72.87), and France (69.37) are higher, indicating that these countries play a pivotal role in information exploration and influence dissemination in this field.

A network consisting of 77 countries was generated using VOSviewer (Figure 2B). The USA and China exhibit the highest level of cooperation. In addition, the USA often collaborates with Italy, South Korea, and the United Kingdom, while China frequently works closely with Japan, Australia, and the United Kingdom. The predominance of the North American, European, and Asian regions in the publications indicates that they are the dominant players (Figure 2C). The links between the USA and other countries are both abundant and bold, signifying a strong awareness of cooperation (Figure 2D).

The total citations of the USA are 71,088 (Figure 2E), far ahead of the United Kingdom (17,825). The H-index of the USA is the highest (120), indicating that the USA has a higher quality of publications in this field (Figure 2F). In conclusion, the USA is leading in terms of academic standards and influence in the field of AT and NAFLD.

### 3.3 Analysis of authors and co-cited authors

The parameter setting for the author collaboration network in CiteSpace remained unchanged, except for years per slice (8), node types (author), and Top N (100). The number of researchers involved in AT and NAFLD has increased over time, with a particular concentration in the last 5 years (Supplementary Figure S1A).

Kenneth Cusi (37 articles) was the most published author, followed by Amalia Gastaldelli (30 articles) and Michael Roden (28 articles). Kenneth Cusi was more active around 2014 (Supplementary Figure S1D), with the highest H-index of 32 (Supplementary Figure S1E). Amalia Gastaldelli mainly published articles around 2017. Michael Roden has mostly published in the last 5 years. The authors are divided into different colored clusters (Supplementary Figure S1C), and thicker lines between authors in the same cluster may indicate a long-term stable collaboration.

The parameter settings for co-cited authors in CiteSpace remained unchanged, except for years per slice (1), selection criteria (k = 20), and pruning (pathfinder), while other parameters remained identical (Supplementary Figure S1B). The top 10 authors were all co-cited more than 400 times (Supplementary Figure S1F). The most co-cited author is Zobair M Younossi (580), followed by Pablo Angulo (551) and Giulio Marchesini (528).

### 3.4 Analysis of journals

**FIGURE 3:**
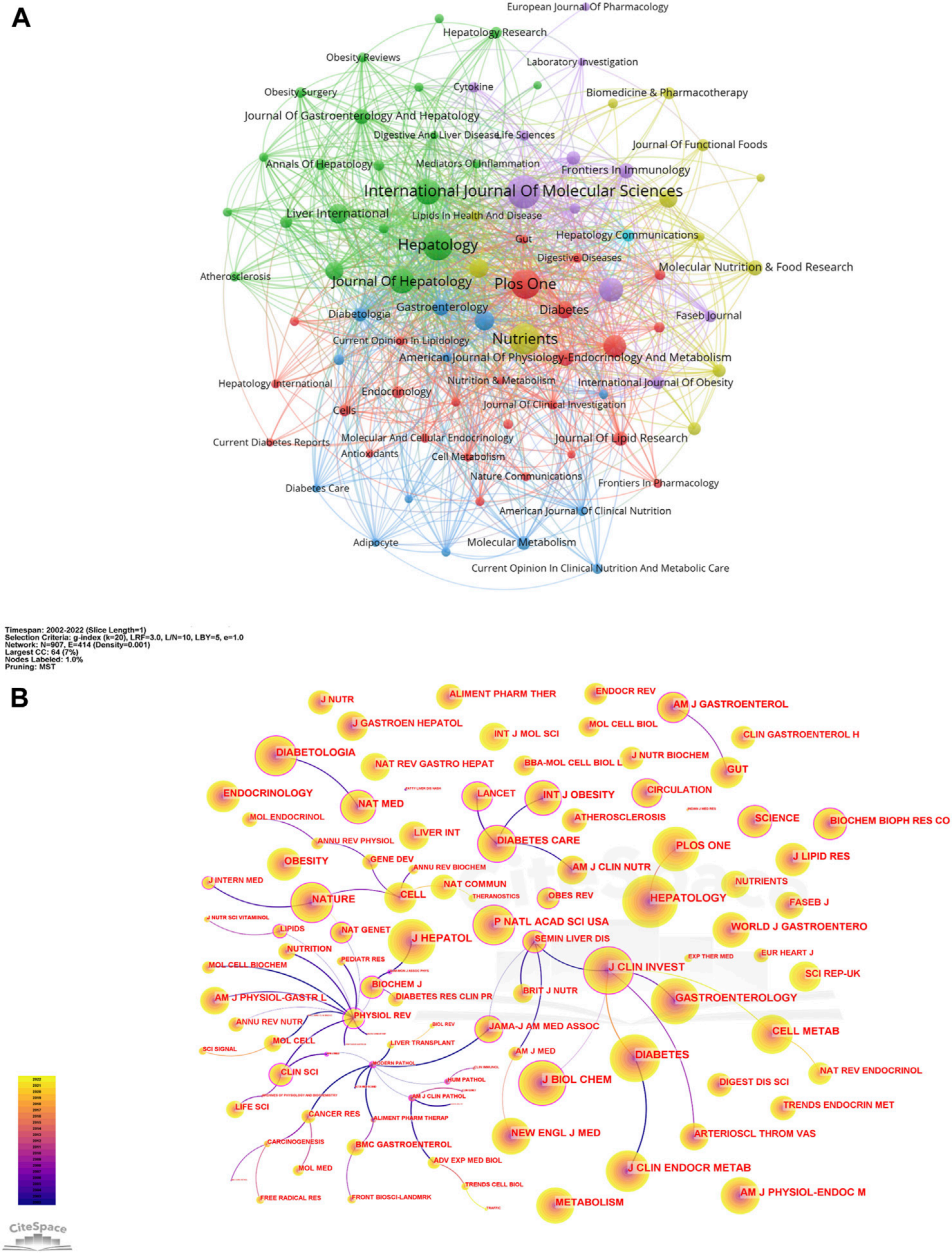
Network of journals and co-cited journals in AT and NAFLD. **(A)** Visual map among journals based on VOSviewer. **(B)** Visual map among co-cited journals based on CiteSpace.

**FIGURE 4:**
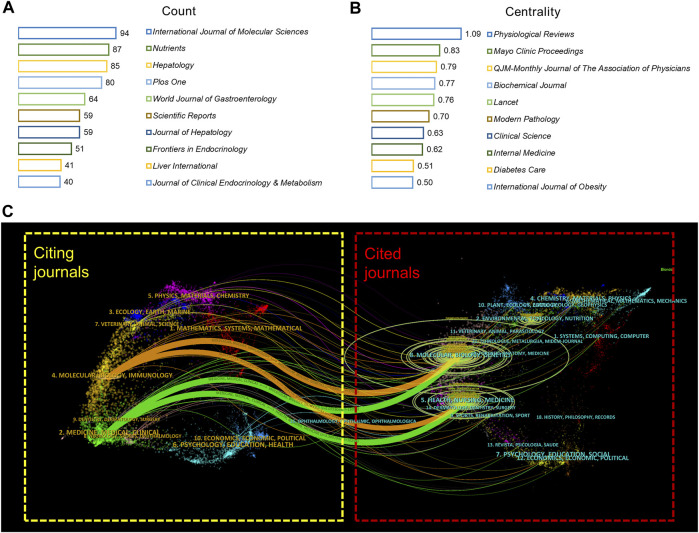
Visual analysis of journals in AT and NAFLD. **(A)** Top 10 journals in terms of publications. **(B)** Top 10 co-cited journals in terms of centrality. **(C)** Dual-map overlay of journals. The citing journals are on the left and the cited journals are on the right, with colored curves representing the citation relationship between them. There are four major curves in this figure, two green paths and two orange paths. The ellipses in the graph represent the number of papers corresponding to the discipline. The longer the horizontal axis of the ellipse, the more authors in that discipline, and the longer the vertical axis of the ellipse, the more papers in that discipline. The f-value represents the frequency of citations, and the z-value is a standardization score of the f-value.

A visual analysis of the sources of articles using VOSviewer (Figure 3A) shows that the *International Journal of Molecular Sciences* (94) had the most publications in AT and NAFLD, followed by *Nutrients* (87) and *Hepatology* (85) (Figure 4A). The top ten journals are all in the Quartile 1 (Q1)/Quartile 2 (Q2) Journal Citation Report (JCR) region, with an H-index above 100, suggesting that they are key journals in the field due to their high disciplinary visibility and publication quality. They can be prioritized for research on AT and NAFLD.

The visualization of co-cited journals was carried out with the following CiteSpace parameters: selection criteria (k = 20), pruning (minimum spanning tree), and other parameters unchanged (Figure 3B). *Hepatology* was the most cited journal (2,625), followed by *Diabetes* (2,239) and the *Journal of Clinical Investigation* (2,072). There were 48 journals with a centrality greater than 0.1 (5.29%), with *Physiological Reviews* (1.09) having the highest centrality, followed by *Mayo Clinic Proceedings* (0.83) and *QJM*-*Monthly Journal of The Association of Physicians* (0.79), indicating the high academic standing and influence of these journals in the field (Figure 4B).

The citing journals in the orange path are related to Molecular/Biology/Immunology, while the cited journals are from (1) Molecular, Biology, Genetics (z = 6.039159, f = 17,828); (2) Health, Nursing, Medicine (z = 2.4764757, f = 7,750). The citing journals in the green path are related to Medicine/Medical/Clinical, and the cited journals are from (1) Molecular, Biology, Genetics (z = 5.0723066, f = 15,093); (2) Health, Nursing, Medicine (z = 3.3492944, f = 10,219). The research studies on AT and NAFLD focused on molecular biology, health management, and life science (Figure 4C). Additionally, the disciplines of Veterinary/Animal/Science and Physics/Materials/Chemistry are also involved in the study of AT and NAFLD, indicating a cross-collaboration between multiple disciplines. The dual-map overlay of journals predicts that hotspots and trends of AT and NAFLD will converge in the fields of Molecular/Biology/Immunology.

### 3.5 Analysis of institutions and co-cited references

The CiteSpace parameters remained unchanged, and a network of 491 nodes and 2,367 links was obtained (Supplementary Figure S2A). The top three institutions were Institut National de la Sante et de la Recherche Medicale, University of California System, and Harvard University, with 106, 105, and 93 publications, respectively, (Supplementary Figure S2B).

**FIGURE 5:**
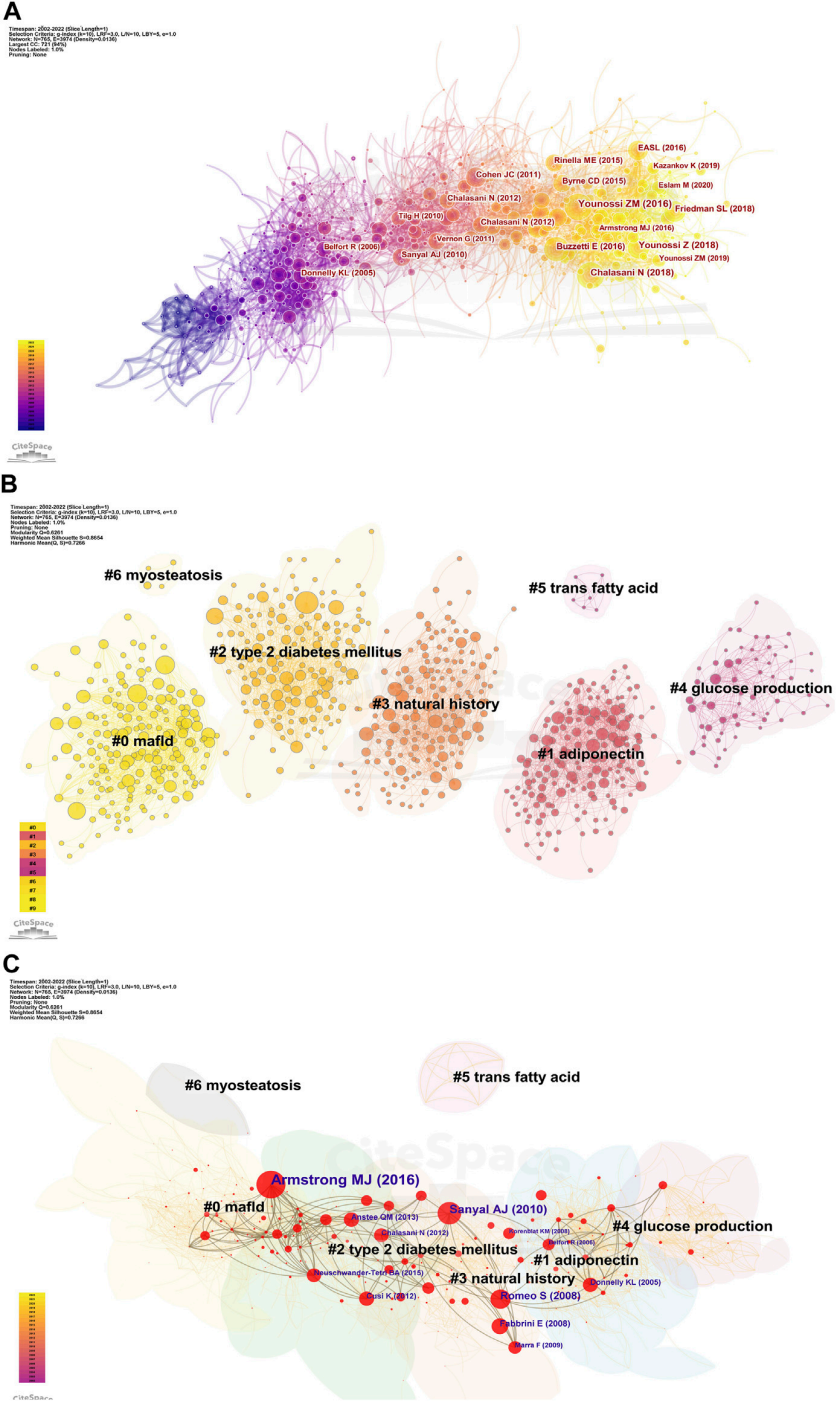
Visualization of co-cited references in AT and NAFLD. **(A)** Reference co-citation network based on CiteSpace. **(B)** Cluster analysis of co-cited references based on CiteSpace. In general, Q and S range from −1 to 1, with higher values indicating tighter clustering and better results. **(C)** Cluster-based hub map of key references.

Next, we analyzed the cited references for co-citation, which provided a more comprehensive interpretation of the role played by AT in NAFLD. The CiteSpace parameters were set as follows: selection criteria (k = 10), and the others remained unchanged. A network with 765 nodes, 3,974 links, and a density of 0.0136 was obtained (Figure 5A). The top 20 references are shown, with 12 published after 2015 and only two before 2010. Nine articles with a centrality of 0.1 or more (Table 2), all from notable journals in the field, are representative works that provide insights into the relevance of AT and NAFLD.

**TABLE 2:** Top 10 co-cited references in terms of centrality in AT and NAFLD.

Label	Year	Centrality	Record count	Title	Source	DOI	Cluster
Armstrong MJ (2016)	2016	0.19	64	Liraglutide safety and efficacy in patients with non-alcoholic steatohepatitis (LEAN): a multicentre, double-blind, randomised, placebo-controlled phase 2 study	Lancet	10.1016/S0140-6736(15)00803-X	0
Sanyal AJ (2010)	2010	0.16	65	Pioglitazone, vitamin E, or placebo for nonalcoholic steatohepatitis	New England Journal of Medicine	10.1056/NEJMoa0907929	3
Romeo S (2008)	2008	0.14	49	Genetic variation in PNPLA3 confers susceptibility to nonalcoholic fatty liver disease	Nature Genetics	10.1038/ng.257	3
Fabbrini E (2008)	2008	0.11	36	Alterations in adipose tissue and hepatic lipid kinetics in obese men and women with nonalcoholic fatty liver disease	Gastroenterology	10.1053/j.gastro. 2007.11.038	3
Chalasani N (2012)	2012	0.1	72	The diagnosis and management of non-alcoholic fatty liver disease: practice Guideline by the American Association for the Study of Liver Diseases, American College of Gastroenterology, and the American Gastroenterological Association	Hepatology	10.1002/hep.25762	3
Donnelly KL (2005)	2005	0.1	66	Sources of fatty acids stored in liver and secreted via lipoproteins in patients with nonalcoholic fatty liver disease	Journal of Clinical Investigation	10.1172/JCI200523621	1
Cusi K (2012)	2012	0.1	56	Role of obesity and lipotoxicity in the development of nonalcoholic steatohepatitis: pathophysiology and clinical implications	Gastroenterology	10.1053/j.gastro. 2012.02.003	2
Anstee QM (2013)	2013	0.1	50	Progression of NAFLD to diabetes mellitus, cardiovascular disease or cirrhosis	Nature Reviews Gastroenterology & Hepatology	10.1038/nrgastro. 2013.41	2
Neuschwander-Tetri BA (2015)	2015	0.1	48	Farnesoid X nuclear receptor ligand obeticholic acid for non-cirrhotic, non-alcoholic steatohepatitis (FLINT): a multicentre, randomised, placebo-controlled trial	Lancet	10.1016/S0140-6736(14)61933-4	2
Marra F (2009)	2009	0.09	35	Adipokines in liver diseases	Hepatology	10.1002/hep.23046	3

We then performed cluster analysis of the references. The clustering results showed Q = 0.6261 and a mean value of S = 0.8654, illustrating a good effect (Figure 5B). A total of 43 clusters were yielded, with seven clusters having more than two nodes. They can be categorized into three main areas. First, pathological states associated with NAFLD include #0 mafld, #2 type 2 diabetes mellitus, and #6 myosteatosis. Second, physiological processes associated with the role of AT in NAFLD include #1 adiponectin, #4 glucose production, and #5 trans-fatty acid. Finally, the evolutionary history of the development of NAFLD and AT was represented by #3 natural history.

The k-cores of the clusters were set at 25, and the strength of the association of key references (in red) can be seen within the same cluster or between different clusters (Figure 5C). Early studies were relatively homogeneous, focusing on glucose production and adiponectin. However, as researchers delved deeper into the field, the themes became more diverse and intersectional, suggesting that studies are becoming more comprehensive in AT and NAFLD. The top 10 cross-thematic references were listed for centrality and will be discussed later (Table 3).

**TABLE 3:** Top 10 cross-thematic references in terms of centrality in AT and NAFLD.

Rank	Centrality	Title	Year	Source	DOI	Cluster
1	0.19	Liraglutide safety and efficacy in patients with non-alcoholic steatohepatitis (LEAN): a multicentre, double-blind, randomised, placebo-controlled phase 2 study	2016	Lancet	10.1016/S0140-6736(15)00803-X	#3, #0
2	0.16	Pioglitazone, vitamin E, or placebo for nonalcoholic steatohepatitis	2010	New England Journal of Medicine	10.1056/NEJMoa0907929	#1, #2, #3
3	0.14	Genetic variation in PNPLA3 confers susceptibility to nonalcoholic fatty liver disease	2008	Nature Genetics	10.1038/ng.257	#1, #3
4	0.10	Sources of fatty acids stored in liver and secreted via lipoproteins in patients with nonalcoholic fatty liver disease	2005	Journal of Clinical Investigation	10.1172/JCI200523621	#1, #4, #3
5	0.10	Role of obesity and lipotoxicity in the development of nonalcoholic steatohepatitis: pathophysiology and clinical implications	2012	Gastroenterology	10.1053/j.gastro. 2012.02.003	#2, #3, #0
6	0.10	Progression of NAFLD to diabetes mellitus, cardiovascular disease or cirrhosis	2013	Nature Reviews Gastroenterology Hepatology	10.1038/nrgastro. 2013.41	#2, #3, #0
7	0.10	Farnesoid X nuclear receptor ligand obeticholic acid for non-cirrhotic, non-alcoholic steatohepatitis (FLINT): a multicentre, randomised, placebo-controlled trial	2015	Lancet	10.1016/S0140-6736(14)61,933-4	#2, #0
8	0.08	A placebo-controlled trial of pioglitazone in subjects with nonalcoholic steatohepatitis	2006	New England Journal of Medicine	10.1056/NEJMoa060326	#1, #3
9	0.08	Evolution of inflammation in nonalcoholic fatty liver disease: the multiple parallel hits hypothesis	2010	Hepatology	10.1002/hep.24001	#3, #2
10	0.08	Exome-wide association study identifies a TM6SF2 variant that confers susceptibility to nonalcoholic fatty liver disease	2014	Nature Genetics	10.1038/ng.2901	#2, #3, #0

### 3.6 Keyword analysis

**FIGURE 6:**
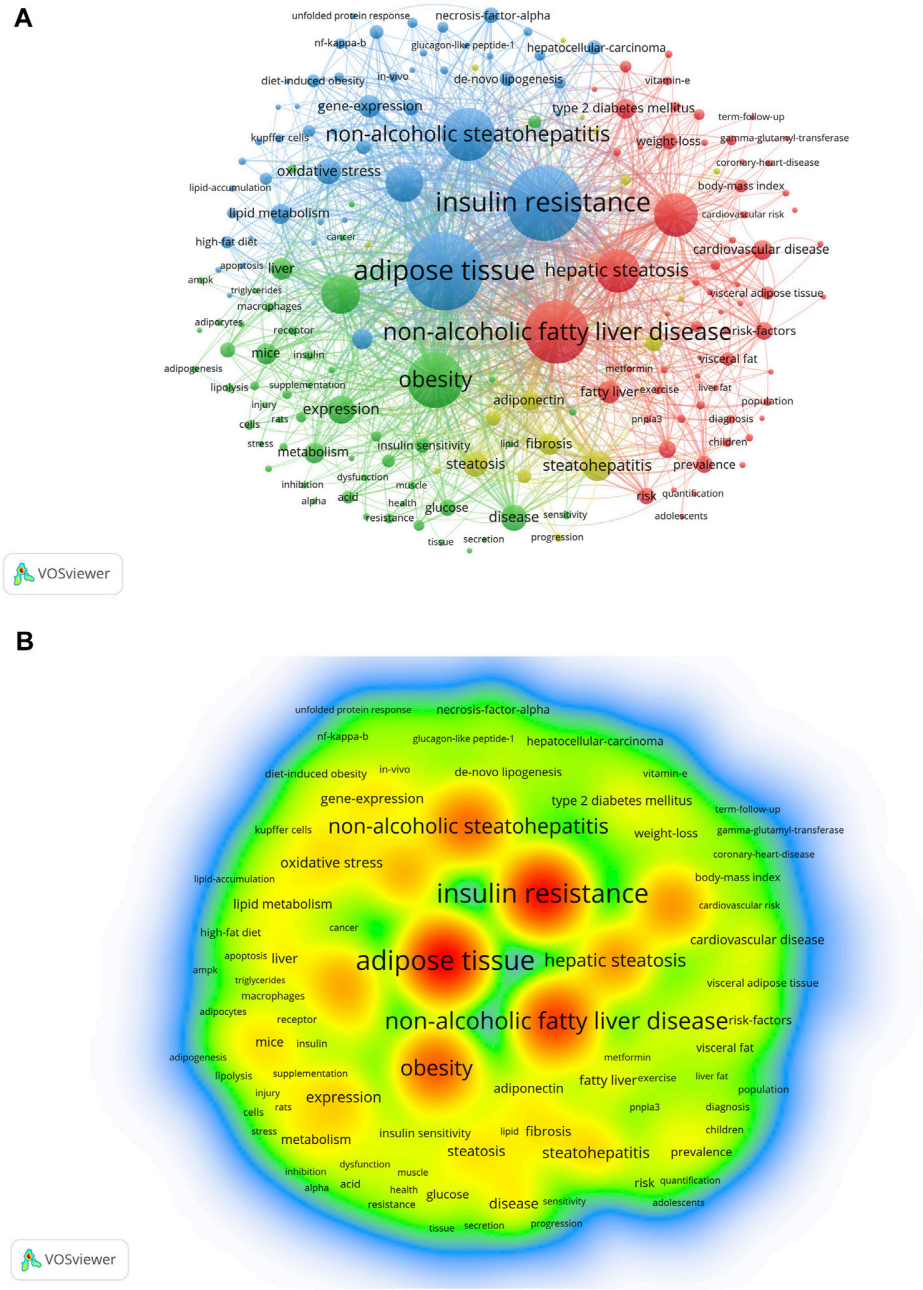
Visualization of keywords in AT and NAFLD based on VOSviewer. **(A)** Visual map of network based on VOSviewer. **(B)** Density visualization based on VOSviewer.

Analysis and statistics of keywords facilitate our understanding of the hot topics and frontiers of a discipline. The keyword network diagram created using VOSviewer contains 8,853 keywords, of which 183 met the threshold. A network (Figure 6A) and density visualization (Figure 6B) were acquired. Unsurprisingly, the keywords with the highest occurrence were adipose tissue and non-alcoholic fatty liver disease, followed by non-alcoholic steatohepatitis, IR, obesity, inflammation, and metabolic syndrome, each with more than 500 occurrences (Table 4).

**TABLE 4:** Top 10 keywords in terms of occurrence in AT and NAFLD.

Rank	Keyword	Occurrence	Total link strength	Rank	Keyword	Occurrence	Total link strength
1	Adipose tissue	1,733	11,468	11	Expression	360	2,414
2	Insulin resistance	1,645	11,338	12	Disease	319	2,186
3	Non-alcoholic fatty liver disease	1,271	8,982	13	Oxidative stress	312	2,192
4	Obesity	1,059	7,408	14	Steatosis	307	2,201
5	Non-alcoholic steatohepatitis	967	6,511	15	Fatty liver	268	1927
6	Metabolic syndrome	721	5,135	16	Gene expression	264	1764
7	Hepatic steatosis	713	4,967	17	Fibrosis	256	1849
8	Inflammation	602	4,380	18	Liver	254	1769
9	Fatty liver-disease	575	3,741	19	Mice	242	1,619
10	Steatohepatitis	410	2,972	20	Metabolism	230	1,573

**FIGURE 7:**
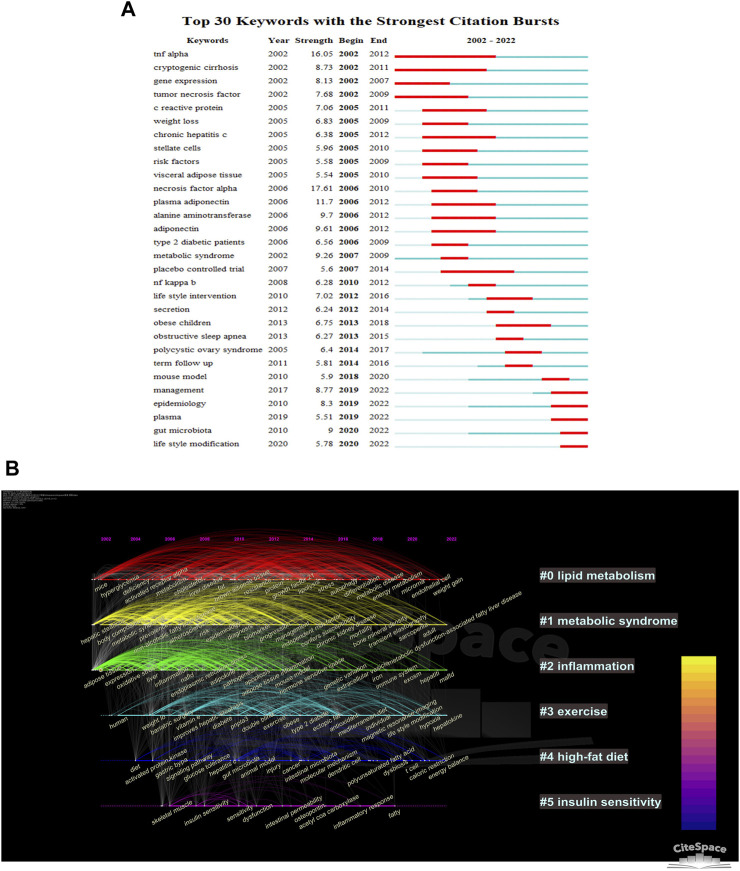
Visualization of keywords in AT and NAFLD based on CiteSpace. **(A)** Top 30 keywords with the strongest citation bursts. The blue line indicates the timeline, with the bolded blue segment indicating the beginning and ending years of the keyword, while the red segment indicates its burst duration period. **(B)** Cluster-based timeline diagram of keywords.

The CiteSpace parameters were set as follows: selection criteria (k = 10), and the others remained unchanged. The results suggest that the risk of obstructive sleep apnea, cryptogenic cirrhosis, polycystic ovary syndrome, and chronic hepatitis may be associated with NAFLD (Figure 7A). The importance of TNF-α is evident from its high strength and continued scholarly attention, despite being researched in the early days. Gut microbiota and lifestyle modification are keywords that are currently experiencing a burst in the field and are likely to remain a focus for researchers in the future.

There are seven clusters presented in Figure 7B, with six of them having more than two nodes. The main manifestations are in three areas. First, metabolic processes associated with NAFLD include #0 lipid metabolism and #1 metabolic syndrome. Second, pathological factors associated with NAFLD include #2 inflammation and #5 insulin sensitivity. Finally, lifestyle factors affecting NAFLD include #3 exercise and #4 high-fat diet. It is evident that lipid metabolism has been widely discussed from the early days to the present. Metabolic syndrome and inflammation received high attention during 2002–2012. Keywords related to exercise and high-fat diet emerged later and have remained in the spotlight to date, with a high level of association between the keywords. However, keywords related to insulin sensitivity only experienced a burst during 2006–2020, with a small number and poor connectivity. The above results suggest that lipid metabolism, metabolic syndrome, exercise, and high-fat diet are current hotspots in AT and NAFLD and will likely remain so in the next 5 years.

## 4 Discussion

### 4.1 General information

In this study, we conducted a literature review on AT and NAFLD from 2002 to 2022. The relevant articles were searched in the WoSCC database, resulting in 3,330 articles after screening and de-duplication. Although only seven papers were published in 2002, the number of publications and citations has steadily increased over the past 20 years. Notably, there was a significant surge in the number of publications after 2008, indicating a growing interest among researchers in this field. This increase can be attributed to the increased financial support for AT and NAFLD research from governments and foundations, as well as the rapid advancements in science and technology during this period. However, it is worth mentioning that the number of publications in 2022 showed a decrease instead of an increase. This decline may be attributed to the implementation of regulations and restrictive policies related to the ongoing epidemic, which have disrupted scientific work in many laboratories.

The primary countries leading the research on AT and NAFLD are the USA, China, and Italy. The USA stands out with the highest centrality and H-index among these nations. Within the top 10 authors, three are from the USA while two are from Italy. When considering the top ten institutions by the number of publications, six originate from the USA. Furthermore, prominent American institutions in this area include Veterans Health Administration (VHA) with the highest H-index, the University of California System with the highest centrality, and the University of Texas System with the highest times cited and average article citations. As evident, the USA holds a leading position in the quality and academic level of research in AT and NAFLD, with an extremely important referential value and guiding role in the academic community. The close communication among countries and institutions in this area has significantly contributed to the promotion of the in-depth development of AT and NAFLD.

Kenneth Cusi holds the top position with 37 publications and an H-index of 32. He reviewed the effects of AT function in NAFLD and pointed out that inhibition of lipolysis and promotion of fatty acid oxidation (FAO) are effective ways to reduce NAFLD (Cusi et al., 2007; Cusi, 2012). His latest article suggests that the AT insulin resistance index (Adipo-IR) plays a more significant role in advanced liver fibrosis in NAFLD than the insulin resistance index (HOMA-IR). Herbert Tilg, with the highest average article citations (198.5), highlighted that adiponectin secreted by AT has anti-inflammatory effects. It can protect the liver from inflammation by inhibiting the expression of TNF-α and IL-6, reducing IR, and thereby improving NAFLD (Tilg, 2010). All of the aforementioned studies suggest that targeting AT is a crucial therapeutic approach to improving hepatic steatosis, inflammation, and liver fibrosis in patients with NAFLD (Kalavalapalli et al., 2023).

Among the top 10 journals, both in terms of publication quantity and co-citations, seven and eight of them, respectively, belong to the Q1 JCR region. This demonstrates the strength of these journals in publication quality, academic standing, and high interest in the domain of AT and NAFLD. The *Journal of Hepatology*, leading the field with the highest impact factor (IF) of 30.083, is a publication dedicated to the medical specialties of gastroenterology and hepatology. The understanding of these literature sources will decidedly help guide future researchers in choosing apt journals to publish their work related to AT and NAFLD.

### 4.2 Key references

Evidently, NAFLD is closely related to factors such as disorders of lipid metabolism, IR, and inflammation. According to cited references and citing references, we summarized the mechanisms of AT-based regulation of NAFLD, with the main focus on inhibiting lipolysis, improving insulin sensitivity, regulating adipokines, and downregulating AT inflammation.

In the first place, the regulation of NAFLD involves lipolysis and FFA-related pathological processes. Donnelly et al. (2005) discovered that lipolysis in AT results in an elevation of peripheral FFA and *de novo* lipogenesis (DNL), which further contributes to hepatic lipid accumulation. Inhibition of lipolysis is suggested to be effective in mitigating the progression of NAFLD. Additionally, disorders of lipid metabolism are significantly related to the intake of dietary FFA. Insufficient ω-3 polyunsaturated fatty acids (PUFA) in the liver are currently one of the important pathogenic triggers of NAFLD. This may be attributed to the decrease in ω-3 PUFA content in AT, limiting the synthesis of ω-3 PUFA in the liver, thereby resulting in an increase in FFA synthesis and a decrease in FAO (Araya et al., 2004). It is suggested that NAFLD can be alleviated by adequate intake of ω-3 PUFA in foods such as deep-sea fish, shellfish, and nuts.

Moreover, the modulation of adipokines has shown to improve IR and NAFLD. AT consists of a diverse array of cells, with adipocytes being the most abundant cell type, accounting for approximately 70% of the tissue. These adipocytes secrete various adipokines, including a multitude of cytokines (inflammatory factors, chemokines, growth factors, etc.), hormones (adiponectin, leptin, resistin, etc.), enzymes, and peptides (Halberg et al., 2008; Lumeng and Saltiel, 2011). For example, increased adiponectin induces an inward flow of extracellular calcium ions, activating adenosine monophosphate (AMP)-activated protein kinase (AMPK) and sirtuin 1 (SIRT1). This activation improves IR, thus preventing hepatic steatosis and fibrosis (Iwabu et al., 2010; Tilg and Moschen, 2010; Cusi, 2012; Shih et al., 2021). Leptin, on the other hand, has the ability to promote lipid oxidation and reduce hepatic triglyceride (TG) accumulation. Conversely, a decrease in resistin inhibits the AMPK/peroxisome proliferator-activated receptor-gamma co-activator 1 alpha (PGC-1α) pathway, leading to the downregulation of pro-inflammatory factors and suppression of liver inflammation (Wen et al., 2021). The aforementioned evidence collectively suggests that the prevention and therapy of NAFLD can begin with the regulation of adipokines.

Finally, the pathogenesis of NAFLD, marked by inflammation in AT, has been explored. Zobair M Younossi hypothesized that AT inflammation precedes liver inflammation in the development of NAFLD (Tilg and Moschen, 2010; Younossi et al., 2010). Studies have demonstrated that AT-secreted TNF-α and IL-6 promote IR and exacerbate NAFLD progression by upregulating the expression of suppressor of cytokine signaling 3 (SOCS3) (Tilg and Moschen, 2010). TNF-α has also been found to disrupt AT function and accelerate NAFLD progression through several pathways, including inducing adipocyte differentiation and proliferation, promoting FFA release, and worsening IR, as well as inhibiting FAO and inducing oxidative stress (Xu et al., 2003). Additionally, Leon A Adams et al. discovered that the inflammation and expansion of visceral adipose tissue (VAT) contribute to the increased risk of incidence of T2DM and CVD associated with NAFLD (Anstee et al., 2013; Adams et al., 2017; Muzurović et al., 2021). It is clear that inflammation in AT poses significant risks. The urgency to target macrophages and immune cells in AT to alleviate NAFLD has been emphasized (Peiseler et al., 2022).

### 4.3 Hotspots and frontiers

Combining and analyzing the terms of high occurrence and the keywords in recent years, we have summarized the research hotspots and frontiers in the field of AT and NAFLD, which mainly include the following three aspects.

#### 4.3.1 Lipid metabolism

##### 4.3.1.1 Lipolysis is a key idea for targeting AT in the treatment of NAFLD

Disorders of systemic lipid metabolism are widely recognized as a major characteristic of NAFLD. The principal pathway of AT involved in lipid metabolism is lipolysis, which is regulated by a variety of factors and mechanisms, including microRNA, AT autophagy, and gut microflora. It has been revealed that miR199a-5p further inhibits AMPK/sterol regulatory element-binding protein-1c (SREBP1c), a key signal for lipolysis, by downregulating the expression of its target gene mammalian STE20-like kinase-1 (MST1), thereby exacerbating hepatic lipid accumulation (Li et al., 2020). Lipolysis is also influenced by white adipose tissue (WAT) autophagy. Specifically inhibiting AT autophagy in mice fed with a high-fat diet (HFD) resulted in increased lipid storage in WAT, decreased lipolysis, and reduced flow of FFA into the liver, contributing to the improvement of hepatic steatosis and liver injury (Sakane et al., 2021). Notably, gut microflora can also modulate lipolysis. *Faecalibacterium prausnitzii*, considered a major marker of intestinal health, constitutes 5%–15% of the total human intestinal bacteria (Hornef and Pabst, 2016). Treatment with *F. prausnitzii* strains LC49 and LB8 significantly elevated acetate levels, effectively inhibiting lipolysis and promoting WAT browning (Sahuri-Arisoylu et al., 2016; Hu et al., 2022). This inhibition of lipolysis in AT emerges as a crucial therapeutic approach in addressing NAFLD, as indicated by the aforementioned studies.

##### 4.3.1.2 Brown adipose tissue-driven energy metabolism is emerging as a research trend in NAFLD intervention

Brown adipose tissue (BAT) is a core organ that regulates energy metabolism and is rich in mitochondria. In recent years, the induction of brown adipocyte differentiation and WAT browning has been recognized as critical factors in preventing and treating NAFLD. Among these factors, inhibition of autophagy represents a relatively new research direction. Studies have shown that the loss of autophagy in AT prevents the degradation of key proteins regulating brown adipocyte differentiation and induces WAT browning (Singh et al., 2009). Unexpectedly, recent findings indicate that mitochondrial autophagy acts in contrast to general autophagy in regulating BAT and systemic energy metabolism. BAT-specific deletion of PTEN-induced kinase I (PINK1), a gene involved in mitochondrial autophagy, induces the differentiation of brown adipocyte precursors (BAPs) into white-like adipocytes, resulting in BAT dysfunction (Ko et al., 2021). In conclusion, while the importance of BAT intervention in NAFLD through energy metabolism is well established, further exploration of the specific regulatory roles and mechanisms affecting AT function based on autophagy is warranted.

##### 4.3.1.3 Regulation of hypoxia is a new direction to improve abnormal AT lipid metabolism in NAFLD.

Hypoxia signaling is one of the key mechanisms of AT lipid disorders. A negative correlation between lipolysis and WAT oxygen tension has been shown in animal models (Pasarica et al., 2010). Hypoxia-inducible transcription factors (HIFs) are the main regulators of cellular and tissue responses to hypoxic stress. Knockout of HIF-1α in adipocyte has been found to have a preventive effect against NAFLD by increasing FFA oxidative metabolism and reducing gluconeogenesis, thereby promoting energy expenditure and improving insulin sensitivity (Jiang et al., 2011). Thus, modulation of the hypoxic response could be a potential target for the treatment of NAFLD. Therefore, Yanfei Zhang et al. demonstrated for the first time that hypoxia induces ferroptosis in AT. Ferroptosis is associated with the progression of oxidative stress and lipid peroxidation in NAFLD. Glutathione peroxidase 4 (GPX4) plays a pivotal role in inhibiting ferroptosis by scavenging lipid peroxidation (Tong et al., 2022; Zhang et al., 2022). Under hypoxic conditions, iron homeostasis in WAT and BAT is dysregulated, leading to a significant decrease in GPX4 expression in subcutaneous WAT and interscapular BAT, which exacerbates lipid peroxidation. Therefore, abnormal AT lipid metabolism caused by hypoxia may serve as a potential triggering mechanism for NAFLD.

##### 4.3.1.4 NAFLD is significantly associated with metabolic diseases

In the present years, NAFLD has also been recognized as metabolic-associated fatty liver disease (MAFLD) due to the presence of various metabolic disorders in patients, including disturbances in lipid, glucose, and insulin metabolism. Therefore, it is not surprising that there is a high correlation between NAFLD and obesity as well as T2DM. The increasing prevalence of NAFLD, driven by the rise in obesity rates, is considered one of the major health crises predicted for the forthcoming decade (Younossi and Henry, 2016). Most obese individuals exhibit excess accumulation of visceral fat and IR, leading to impaired insulin-mediated suppression of lipolysis. Consequently, elevated levels of FFA enter the bloodstream, promoting ectopic lipid deposition and the development of NAFLD. Notably, approximately 75% of patients with T2DM also suffer from NAFLD, and there is a likelihood of 10%–18% that adult NAFLD patients will have concurrent T2DM (Kwok et al., 2016). As NAFLD improves or enters remission, the risk of developing T2DM appears to decrease over time (Fukuda et al., 2016).

It is worth noting that the association between NAFLD and sarcopenia has also gained increased attention in recent years. Sarcopenia, characterized by a progressive loss of skeletal muscle mass, is closely linked to metabolic dysfunction (Zambon Azevedo et al., 2021). The muscle–AT–liver axis has emerged as a potential determinant in the interplay between sarcopenia and NAFLD (Merli et al., 2019). To be specific, adiponectin receptors in muscle play a role in regulating insulin signaling, enhancing FFA oxidation, and reducing DNL. Conversely, the muscle growth inhibitor, signaling proteins secreted by muscle cells, contributes to increased AT mass and decreased adiponectin secretion. In summary, metabolic disorders, including sarcopenia, serve as adverse occurrences or prognostic factors for individuals with NAFLD. Therefore, timely identification and preventive measures are of utmost importance.

#### 4.3.2 Inflammatory mechanisms in AT of NAFLD

##### 4.3.2.1 Role of AT inflammation in NAFLD progression

Previous basic and clinical studies have confirmed that AT inflammation is associated with immune cell recruitment and the release of adipokines. Specifically, during the development of NAFLD, AT secretes increased levels of pro-inflammatory factors, which are upregulated by the stimulation of resistin and leptin. Meanwhile, the levels of anti-inflammatory factors and adipokines, which inhibit the secretion of pro-inflammatory factors, decrease (du Plessis et al., 2015; Shih et al., 2021). Additionally, the activation and recruitment of T cells within AT play a crucial role in WAT inflammation. Research has shown that inhibiting Th17 and cytotoxic T (Tc) among T-cell subsets in VAT, along with activating Treg, can reduce plasma ALT and leptin levels, alleviating hepatic steatosis, inflammation, and fibrosis, thereby improving NAFLD (He et al., 2017; Van Herck et al., 2020). Adipocyte-derived miR-326 directly participates in the polarization of Th1 cells to Th17 cells, triggering AT inflammation (Kiran et al., 2021). In summary, modulating AT inflammation plays a significant role in the treatment of NAFLD.

##### 4.3.2.2 Hepatokine is an important regulator of NAFLD

Analogous to adipokines and myokines, hepatokines are proteins secreted by hepatocytes with paracrine and/or endocrine activity. This class of organic factors has garnered increased attention in recent years concerning the pathogenesis of metabolic syndrome, T2DM, CVD, and NAFLD (Ren et al., 2022). The liver appears to regulate AT inflammation and impacts lipid metabolism through the release of hepatokines into the bloodstream. Fetuin-A (Fet-A), the first hepatokine discovered to have a significant pathogenic effect in NAFLD, facilitates the presentation of FFA to toll-like receptor 4 (TLR4) by inducing AT macrophage infiltration and M1 polarization, thereby triggering a series of inflammatory responses (Cayatte et al., 1990). Recently, fibroblast growth factor 21 (FGF21) has also been identified as a crucial hepatokine that is involved in regulating AT inflammation. A study demonstrated that FGF21 effectively prevented the formation of crown-like structures (CLS) induced by a high-fat high-cholesterol diet (HFCD) and inhibited the expression of adhesion G-protein-coupled receptor E1 (Adgre1), along with macrophage infiltration in gonadal WAT (Liu et al., 2023). In summary, the exploration of hepatokines contributes to a deeper understanding of the role of AT–liver crosstalk in the pathogenesis of NAFLD.

#### 4.3.3 Lifestyle modification

##### 4.3.3.1 Lifestyle modification effectively manages the progression of NAFLD

Lifestyle changes, including a sensible diet and exercise therapy, are currently considered the most effective interventions for managing NAFLD. Various dietary factors, such as fructose, trans-fat, and cholesterol, have been demonstrated to contribute to the development of NAFLD/NASH through different mechanisms. Fructose, in addition to promoting fat accumulation by stimulating DNL, is also believed to induce inflammation and endoplasmic reticulum stress, leading to NAFLD (Sapp et al., 2014). Trans-fats, commonly found in snacks, baked goods, and fried food, have been shown to induce NASH in sedentary mice, either alone or in combination with a high-fructose diet (Alisi et al., 2011). Dietary intake of cholesterol impairs mitochondrial function, increases the production of reactive oxygen species (ROS), induces endoplasmic reticulum stress, and leads to hepatocyte death (Min et al., 2012). Therefore, avoiding diets rich in these substances is a practical measure to improve NAFLD. Anita M van den Hoek et al. demonstrated a significant improvement in inflammation in WAT when the diet of LDLR−/− mice was changed from high-fat to low-fat. Exercise alone has also been shown to effectively reduce inflammation (van den Hoek et al., 2021). The combination of the two methods almost completely reversed liver steatosis and inflammation. Sports trainings of low or moderate intensity and long duration, such as aerobic and resistance training, is considered the best approach to fat loss (Kolnes et al., 2021). This may be attributed to the fact that exercise leads to elevated concentrations of IL-6 in plasma, which enhances systemic lipolysis and FAO (van Hall et al., 2003). Additionally, exercise is known to increase insulin sensitivity and improve AT function, including enhancing fat storage and oxidation, reducing systemic low-grade inflammation, and preventing or improving NAFLD (Park et al., 2014). The search for a sensible diet and exercise regimen to prevent NAFLD has gained significant attention in recent years due to their substantial efficacy.

##### 4.3.3.2 The MED is considered to be a lifestyle model for the treatment of NAFLD

The Mediterranean diet (MED) is a dietary approach centered around vegetables, fruits, fish, whole grains, legumes, and olive oil. It emphasizes regular exercise and a positive outlook on life, making it an effective and easily manageable dietary pattern for treating NAFLD (Plaz Torres et al., 2019). The MED diet primarily improves NAFLD by reducing VAT accumulation. Zelicha et al. (2022) suggested that the reduction in VAT associated with the MED may be attributed to its polyphenol content. For instance, urolithin A, an anti-obesity compound found in polyphenols, enhances energy expenditure by boosting BAT thermogenesis and promotes browning of WAT. This, in turn, reduces VAT accumulation and contributes to the amelioration of NAFLD (Xia et al., 2020). Another study demonstrated that the Mediterranean/low-carbohydrate (MED/LC) diet significantly lowered hepatic fat content (HFC) compared to the low-fat (LF) diet. HFC appears to be positively associated with the level of vaspin, a VAT-derived factor linked to obesity and impaired insulin sensitivity (Youn et al., 2008; Gepner et al., 2019). These findings highlight that the MED can enhance NAFLD outcomes by mitigating VAT content.

Scientists have invested substantial efforts in unraveling the potential mechanisms of NAFLD induced by AT dysfunction. However, there is a remarkable scarcity of pharmacodynamic studies involving agonists or inhibitors targeting these mechanisms, and large-sample clinical studies are notably lacking. Future collaborations among experts in this field are imperative to conduct more robust *in vitro* mechanistic experiments and clinical investigations, thereby addressing this critical gap.

Moreover, the current definitive diagnostic method for NAFLD involves liver biopsy, which is invasive and carries potential risks to the body. Exploring a new non-invasive approach to diagnose AT-related changes would signify a significant clinical advancement. In the ongoing research, infrared thermography has shown promise in indirectly reflecting BAT function in mice. The crucial question is whether this outcome can be extrapolated to human subjects. Additionally, the utilization of abdominal MRI imaging to assess deep subcutaneous adipose tissue (dSAT) and VAT compartment volume seems to provide insights into hepatic histological changes in patients with NASH (Shen et al., 2023). The assessment of disease progression in patients with NAFLD through the observation or measurement of other AT indicators remains an open question that requires further exploration.

## 5 Conclusion

Through bibliometric analysis, this study provides valuable insights into the impact of AT function on the progression of NAFLD. The findings highlight several current research hotspots in this field, including lipid metabolism, inflammation, and lifestyle modification. Additionally, emerging areas of interest include microRNA, sarcopenia, T cell, hypoxia, hepatokine, gut microbiota, and autophagy. These topics are at the forefront of research and hold great potential for advancing our understanding of AT and NAFLD.

## Data Availability

The datasets presented in this study can be found in online repositories. The names of the repository/repositories and accession number(s) can be found in the article/Supplementary Material.

## References

[B1] Adams L. A. Anstee Q. M. Tilg H. Targher G. (2017). Non-alcoholic fatty liver disease and its relationship with cardiovascular disease and other extrahepatic diseases. Gut 66 (6), 1138–1153. 10.1136/gutjnl-2017-313884 28314735

[B2] Alisi A. Bedogni G. De Vito R. Comparcola D. Manco M. Nobili V. (2011). Relationship between portal chronic inflammation and disease severity in paediatric non-alcoholic fatty liver disease. Dig. Liver Dis. 43 (2), 143–146. 10.1016/j.dld.2010.05.007 20580331

[B3] Anstee Q. M. Targher G. Day C. P. (2013). Progression of NAFLD to diabetes mellitus, cardiovascular disease or cirrhosis. Nat. Rev. Gastroenterol. Hepatol. 10 (6), 330–344. 10.1038/nrgastro.2013.41 23507799

[B4] Araya J. Rodrigo R. Videla L. A. Thielemann L. Orellana M. Pettinelli P. (2004). Increase in long-chain polyunsaturated fatty acid n - 6/n - 3 ratio in relation to hepatic steatosis in patients with non-alcoholic fatty liver disease. Clin. Sci. (Lond) 106 (6), 635–643. 10.1042/cs20030326 14720121

[B5] Byrne C. D. Targher G. (2015). NAFLD: a multisystem disease. J. Hepatol. 62 (1 Suppl. l), S47–S64. 10.1016/j.jhep.2014.12.012 25920090

[B6] Cai D. Yuan M. Frantz D. F. Melendez P. A. Hansen L. Lee J. (2005). Local and systemic insulin resistance resulting from hepatic activation of IKK-beta and NF-kappaB. Nat. Med. 11 (2), 183–190. 10.1038/nm1166 15685173 PMC1440292

[B7] Cayatte A. J. Kumbla L. Subbiah M. T. (1990). Marked acceleration of exogenous fatty acid incorporation into cellular triglycerides by fetuin. J. Biol. Chem. 265 (10), 5883–5888. 10.1016/s0021-9258(19)39445-1 1690716

[B8] Chen C. (2004). Searching for intellectual turning points: progressive knowledge domain visualization. Proc. Natl. Acad. Sci. U. S. A. 101 5303–5310. 10.1073/pnas.0307513100 14724295 PMC387312

[B9] Cusi K. (2012). Role of obesity and lipotoxicity in the development of nonalcoholic steatohepatitis: pathophysiology and clinical implications. Gastroenterology 142 (4), 711–725. 10.1053/j.gastro.2012.02.003 22326434

[B10] Cusi K. Kashyap S. Gastaldelli A. Bajaj M. Cersosimo E. (2007). Effects on insulin secretion and insulin action of a 48-h reduction of plasma free fatty acids with acipimox in nondiabetic subjects genetically predisposed to type 2 diabetes. Am. J. Physiol. Endocrinol. Metab. 292 (6), E1775–E1781. 10.1152/ajpendo.00624.2006 17299078

[B11] Donnelly K. L. Smith C. I. Schwarzenberg S. J. Jessurun J. Boldt M. D. Parks E. J. (2005). Sources of fatty acids stored in liver and secreted via lipoproteins in patients with nonalcoholic fatty liver disease. J. Clin. Investig. 115(5), 1343–1351. 10.1172/jci23621 15864352 PMC1087172

[B12] du Plessis J. van Pelt J. Korf H. Mathieu C. van der Schueren B. Lannoo M. (2015). Association of adipose tissue inflammation with histologic severity of nonalcoholic fatty liver disease. Gastroenterology 149(3), 635–648.e614. 10.1053/j.gastro.2015.05.044 26028579

[B13] Ellegaard O. Wallin J. A. (2015). The bibliometric analysis of scholarly production: how great is the impact? Scientometrics 105 (3), 1809–1831. 10.1007/s11192-015-1645-z 26594073 PMC4643120

[B14] Estes C. Anstee Q. M. Arias-Loste M. T. Bantel H. Bellentani S. Caballeria J. (2018). Modeling NAFLD disease burden in China, France, Germany, Italy, Japan, Spain, United Kingdom, and United States for the period 2016-2030. J. Hepatol. 69 (4), 896–904. 10.1016/j.jhep.2018.05.036 29886156

[B15] Fukuda T. Hamaguchi M. Kojima T. Mitsuhashi K. Hashimoto Y. Ohbora A. (2016). Transient remission of nonalcoholic fatty liver disease decreases the risk of incident type 2 diabetes mellitus in Japanese men. Eur. J. Gastroenterol. Hepatol. 28 (12), 1443–1449. 10.1097/meg.0000000000000736 27603300

[B16] Ge Y. Chao T. Sun J. Liu W. Chen Y. Wang C. (2022). Frontiers and hotspots evolution in psycho-cardiology: a bibliometric analysis from 2004 to 2022. Curr. Probl. Cardiol. 47 (12), 101361. 10.1016/j.cpcardiol.2022.101361 35995242

[B17] Gepner Y. Shelef I. Komy O. Cohen N. Schwarzfuchs D. Bril N. (2019). The beneficial effects of Mediterranean diet over low-fat diet may be mediated by decreasing hepatic fat content. J. Hepatol. 71 (2), 379–388. 10.1016/j.jhep.2019.04.013 31075323

[B18] Grech V. Rizk D. E. E. (2018). Increasing importance of research metrics: journal Impact Factor and h-index. Int. Urogynecol J. 29 (5), 619–620. 10.1007/s00192-018-3604-8 29549395

[B19] Halberg N. Wernstedt-Asterholm I. Scherer P. E. (2008). The adipocyte as an endocrine cell. Endocrinol. Metab. Clin. North Am. 37 (3), 753–768. x-xi. 10.1016/j.ecl.2008.07.002 18775362 PMC2659415

[B20] He B. Wu L. Xie W. Shao Y. Jiang J. Zhao Z. (2017). The imbalance of Th17/Treg cells is involved in the progression of nonalcoholic fatty liver disease in mice. BMC Immunol. 18 (1), 33. 10.1186/s12865-017-0215-y 28646856 PMC5483270

[B21] Hornef M. W. Pabst O. (2016). Real friends: faecalibacterium prausnitzii supports mucosal immune homeostasis. Gut 65 (3), 365–367. 10.1136/gutjnl-2015-310027 26531718

[B22] Hu W. Gao W. Liu Z. Fang Z. Wang H. Zhao J. (2022). Specific strains of faecalibacterium prausnitzii ameliorate nonalcoholic fatty liver disease in mice in association with gut microbiota regulation. Nutrients 14 (14), 2945. 10.3390/nu14142945 35889903 PMC9325077

[B23] Iwabu M. Yamauchi T. Okada-Iwabu M. Sato K. Nakagawa T. Funata M. (2010). Adiponectin and AdipoR1 regulate PGC-1alpha and mitochondria by Ca(2+) and AMPK/SIRT1. Nature 464 (7293), 1313–1319. 10.1038/nature08991 20357764

[B24] Jiang C. Qu A. Matsubara T. Chanturiya T. Jou W. Gavrilova O. (2011). Disruption of hypoxia-inducible factor 1 in adipocytes improves insulin sensitivity and decreases adiposity in high-fat diet-fed mice. Diabetes 60 (10), 2484–2495. 10.2337/db11-0174 21873554 PMC3178277

[B25] Kalavalapalli S. Leiva E. G. Lomonaco R. Chi X. Shrestha S. Dillard R. (2023). Adipose tissue insulin resistance predicts the severity of liver fibrosis in patients with type 2 diabetes and NAFLD. J. Clin. Endocrinol. Metab. 108 (5), 1192–1201. 10.1210/clinem/dgac660 36378995

[B26] Kiran S. Kumar V. Kumar S. Price R. L. Singh U. P. (2021). Adipocyte, immune cells, and miRNA crosstalk: a novel regulator of metabolic dysfunction and obesity. Cells 10 (5), 1004. 10.3390/cells10051004 33923175 PMC8147115

[B27] Kleiner D. E. Brunt E. M. Van Natta M. Behling C. Contos M. J. Cummings O. W. (2005). Design and validation of a histological scoring system for nonalcoholic fatty liver disease. Hepatology 41 (6), 1313–1321. 10.1002/hep.20701 15915461

[B28] Ko M. S. Yun J. Y. Baek I. J. Jang J. E. Hwang J. J. Lee S. E. (2021). Mitophagy deficiency increases NLRP3 to induce brown fat dysfunction in mice. Autophagy 17 (5), 1205–1221. 10.1080/15548627.2020.1753002 32400277 PMC8143238

[B29] Kolnes K. J. Petersen M. H. Lien-Iversen T. Højlund K. Jensen J. (2021). Effect of exercise training on fat loss-energetic perspectives and the role of improved adipose tissue function and body fat distribution. Front. Physiol. 12, 737709. 10.3389/fphys.2021.737709 34630157 PMC8497689

[B30] Kwok R. Choi K. C. Wong G. L. Zhang Y. Chan H. L. Luk A. O. (2016). Screening diabetic patients for non-alcoholic fatty liver disease with controlled attenuation parameter and liver stiffness measurements: a prospective cohort study. Gut 65 (8), 1359–1368. 10.1136/gutjnl-2015-309265 25873639

[B31] Li Y. Luan Y. Li J. Song H. Li Y. Qi H. (2020). Exosomal miR-199a-5p promotes hepatic lipid accumulation by modulating MST1 expression and fatty acid metabolism. Hepatol. Int. 14 (6), 1057–1074. 10.1007/s12072-020-10096-0 33037981

[B32] Liu C. Schönke M. Spoorenberg B. Lambooij J. M. van der Zande H. J. P. Zhou E. (2023). FGF21 protects against hepatic lipotoxicity and macrophage activation to attenuate fibrogenesis in nonalcoholic steatohepatitis. Elife 12, e83075. 10.7554/eLife.83075 36648330 PMC9928421

[B33] Lumeng C. N. Saltiel A. R. (2011). Inflammatory links between obesity and metabolic disease. J. Clin. Investig. 121 (6), 2111–2117. 10.1172/jci57132 21633179 PMC3104776

[B34] Lv J. Li Y. Shi S. Liu S. Xu X. Wu H. (2023). Frontier and hotspot evolution in cardiorenal syndrome: a bibliometric analysis from 2003 to 2022. Curr. Probl. Cardiol. 48 (8), 101238. 10.1016/j.cpcardiol.2022.101238 35500729

[B35] Ma D. Guan B. Song L. Liu Q. Fan Y. Zhao L. (2021). A bibliometric analysis of exosomes in cardiovascular diseases from 2001 to 2021. Front. Cardiovasc Med. 8, 734514. 10.3389/fcvm.2021.734514 34513962 PMC8424118

[B36] Merli M. Lattanzi B. Aprile F. (2019). Sarcopenic obesity in fatty liver. Curr. Opin. Clin. Nutr. Metab. Care 22 (3), 185–190. 10.1097/mco.0000000000000558 30893090

[B37] Min H. K. Kapoor A. Fuchs M. Mirshahi F. Zhou H. Maher J. (2012). Increased hepatic synthesis and dysregulation of cholesterol metabolism is associated with the severity of nonalcoholic fatty liver disease. Cell. Metab. 15 (5), 665–674. 10.1016/j.cmet.2012.04.004 22560219 PMC3361911

[B38] Musso G. Gambino R. Biroli G. Carello M. Fagà E. Pacini G. (2005). Hypoadiponectinemia predicts the severity of hepatic fibrosis and pancreatic Beta-cell dysfunction in nondiabetic nonobese patients with nonalcoholic steatohepatitis. Am. J. Gastroenterol. 100 (11), 2438–2446. 10.1111/j.1572-0241.2005.00297.x 16279898

[B39] Muzurović E. Mikhailidis D. P. Mantzoros C. (2021). Non-alcoholic fatty liver disease, insulin resistance, metabolic syndrome and their association with vascular risk. Metabolism 119, 154770. 10.1016/j.metabol.2021.154770 33864798

[B40] Park Y. M. Myers M. Vieira-Potter V. J. (2014). Adipose tissue inflammation and metabolic dysfunction: role of exercise. Mo Med. 111 (1), 65–72. 24645302 PMC6179510

[B41] Pasarica M. Rood J. Ravussin E. Schwarz J. M. Smith S. R. Redman L. M. (2010). Reduced oxygenation in human obese adipose tissue is associated with impaired insulin suppression of lipolysis. J. Clin. Endocrinol. Metab. 95 (8), 4052–4055. 10.1210/jc.2009-2377 20466783 PMC2913036

[B42] Peiseler M. Schwabe R. Hampe J. Kubes P. Heikenwälder M. Tacke F. (2022). Immune mechanisms linking metabolic injury to inflammation and fibrosis in fatty liver disease - novel insights into cellular communication circuits. J. Hepatol. 77 (4), 1136–1160. 10.1016/j.jhep.2022.06.012 35750137

[B43] Peng C. Kuang L. Zhao J. Ross A. E. Wang Z. Ciolino J. B. (2022). Bibliometric and visualized analysis of ocular drug delivery from 2001 to 2020. J. Control Release 345, 625–645. 10.1016/j.jconrel.2022.03.031 35321827

[B44] Plaz Torres M. C. Aghemo A. Lleo A. Bodini G. Furnari M. Marabotto E. (2019). Mediterranean diet and NAFLD: what we know and questions that still need to Be answered. Nutrients 11 (12), 2971. 10.3390/nu11122971 31817398 PMC6949938

[B45] Polyzos S. A. Kountouras J. Mantzoros C. S. (2015). Leptin in nonalcoholic fatty liver disease: a narrative review. Metabolism 64 (1), 60–78. 10.1016/j.metabol.2014.10.012 25456097

[B46] Powell E. E. Wong V. W. Rinella M. (2021). Non-alcoholic fatty liver disease. Lancet 397 (10290), 2212–2224. 10.1016/s0140-6736(20)32511-3 33894145

[B47] Ren Y. Zhao H. Yin C. Lan X. Wu L. Du X. (2022). Adipokines, hepatokines and myokines: focus on their role and molecular mechanisms in adipose tissue inflammation. Front. Endocrinol. (Lausanne) 13, 873699. 10.3389/fendo.2022.873699 35909571 PMC9329830

[B48] Sahuri-Arisoylu M. Brody L. P. Parkinson J. R. Parkes H. Navaratnam N. Miller A. D. (2016). Reprogramming of hepatic fat accumulation and 'browning' of adipose tissue by the short-chain fatty acid acetate. Int. J. Obes. (Lond) 40 (6), 955–963. 10.1038/ijo.2016.23 26975441

[B49] Sakane S. Hikita H. Shirai K. Myojin Y. Sasaki Y. Kudo S. (2021). White adipose tissue autophagy and adipose-liver crosstalk exacerbate nonalcoholic fatty liver disease in mice. Cell. Mol. Gastroenterol. Hepatol. 12 (5), 1683–1699. 10.1016/j.jcmgh.2021.07.008 34303881 PMC8551788

[B50] Sapp V. Gaffney L. EauClaire S. F. Matthews R. P. (2014). Fructose leads to hepatic steatosis in zebrafish that is reversed by mechanistic target of rapamycin (mTOR) inhibition. Hepatology 60 (5), 1581–1592. 10.1002/hep.27284 25043405

[B51] Shen W. Middleton M. S. Cunha G. M. Delgado T. I. Wolfson T. Gamst A. (2023). Changes in abdominal adipose tissue depots assessed by MRI correlate with hepatic histologic improvement in non-alcoholic steatohepatitis. J. Hepatol. 78 (2), 238–246. 10.1016/j.jhep.2022.10.027 36368598 PMC9852022

[B52] Shih P. H. Shiue S. J. Chen C. N. Cheng S. W. Lin H. Y. Wu L. W. (2021). Fucoidan and fucoxanthin attenuate hepatic steatosis and inflammation of NAFLD through modulation of leptin/adiponectin Axis. Mar. Drugs 19 (3), 148. 10.3390/md19030148 33809062 PMC8001566

[B53] Singh R. Xiang Y. Wang Y. Baikati K. Cuervo A. M. Luu Y. K. (2009). Autophagy regulates adipose mass and differentiation in mice. J. Clin. Investig. 119 (11), 3329–3339. 10.1172/jci39228 19855132 PMC2769174

[B54] Tilg H. (2010). The role of cytokines in non-alcoholic fatty liver disease. Dig. Dis. 28 (1), 179–185. 10.1159/000282083 20460908

[B55] Tilg H. Moschen A. R. (2010). Evolution of inflammation in nonalcoholic fatty liver disease: the multiple parallel hits hypothesis. Hepatology 52 (5), 1836–1846. 10.1002/hep.24001 21038418

[B56] Tong J. Li D. Meng H. Sun D. Lan X. Ni M. (2022). Targeting a novel inducible GPX4 alternative isoform to alleviate ferroptosis and treat metabolic-associated fatty liver disease. Acta Pharm. Sin. B 12 (9), 3650–3666. 10.1016/j.apsb.2022.02.003 36176906 PMC9513461

[B57] van den Hoek A. M. de Jong J. Worms N. van Nieuwkoop A. Voskuilen M. Menke A. L. (2021). Diet and exercise reduce pre-existing NASH and fibrosis and have additional beneficial effects on the vasculature, adipose tissue and skeletal muscle via organ-crosstalk. Metabolism 124, 154873. 10.1016/j.metabol.2021.154873 34478753

[B58] van der Poorten D. Milner K. L. Hui J. Hodge A. Trenell M. I. Kench J. G. (2008). Visceral fat: a key mediator of steatohepatitis in metabolic liver disease. Hepatology 48 (2), 449–457. 10.1002/hep.22350 18627003

[B59] van Eck N. J. Waltman L. (2010). Software survey: VOSviewer, a computer program for bibliometric mapping. Scientometrics 84 (2), 523–538. 10.1007/s11192-009-0146-3 20585380 PMC2883932

[B60] van Hall G. Steensberg A. Sacchetti M. Fischer C. Keller C. Schjerling P. (2003). Interleukin-6 stimulates lipolysis and fat oxidation in humans. J. Clin. Endocrinol. Metab. 88 (7), 3005–3010. 10.1210/jc.2002-021687 12843134

[B61] Van Herck M. A. Vonghia L. Kwanten W. J. Julé Y. Vanwolleghem T. Ebo D. G. (2020). Diet reversal and immune modulation show key role for liver and adipose tissue T cells in murine nonalcoholic steatohepatitis. Cell. Mol. Gastroenterol. Hepatol. 10 (3), 467–490. 10.1016/j.jcmgh.2020.04.010 32360637 PMC7365964

[B62] Wen F. Shi Z. Liu X. Tan Y. Wei L. Zhu X. (2021). Acute elevated resistin exacerbates mitochondrial damage and aggravates liver steatosis through AMPK/PGC-1α signaling pathway in male NAFLD mice. Horm. Metab. Res. 53 (2), 132–144. 10.1055/a-1293-8250 33302316

[B63] Xia B. Shi X. C. Xie B. C. Zhu M. Q. Chen Y. Chu X. Y. (2020). Urolithin A exerts antiobesity effects through enhancing adipose tissue thermogenesis in mice. PLoS Biol. 18 (3), e3000688. 10.1371/journal.pbio.3000688 32218572 PMC7141696

[B64] Xu H. Barnes G. T. Yang Q. Tan G. Yang D. Chou C. J. (2003). Chronic inflammation in fat plays a crucial role in the development of obesity-related insulin resistance. J. Clin. Investig. 112 (12), 1821–1830. 10.1172/jci19451 14679177 PMC296998

[B65] Youn B. S. Klöting N. Kratzsch J. Lee N. Park J. W. Song E. S. (2008). Serum vaspin concentrations in human obesity and type 2 diabetes. Diabetes 57 (2), 372–377. 10.2337/db07-1045 17991760

[B66] Younossi Z. Henry L. (2016). Contribution of alcoholic and nonalcoholic fatty liver disease to the burden of liver-related morbidity and mortality. Gastroenterology 150 (8), 1778–1785. 10.1053/j.gastro.2016.03.005 26980624

[B67] Younossi Z. M. Baranova A. Stepanova M. Page S. Calvert V. S. Afendy A. (2010). Phosphoproteomic biomarkers predicting histologic nonalcoholic steatohepatitis and fibrosis. J. Proteome Res. 9 (6), 3218–3224. 10.1021/pr100069e 20441224

[B68] Zambon Azevedo V. Silaghi C. A. Maurel T. Silaghi H. Ratziu V. Pais R. (2021). Impact of sarcopenia on the severity of the liver damage in patients with non-alcoholic fatty liver disease. Front. Nutr. 8, 774030. 10.3389/fnut.2021.774030 35111794 PMC8802760

[B69] Zelicha H. Kloting N. Kaplan A. Yaskolka Meir A. Rinott E. Tsaban G. (2022). The effect of high-polyphenol Mediterranean diet on visceral adiposity: the DIRECT PLUS randomized controlled trial. BMC Med. 20 (1), 327. 10.1186/s12916-022-02525-8 36175997 PMC9523931

[B70] Zhang Y. Fang J. Dong Y. Ding H. Cheng Q. Liu H. (2022). High-altitude hypoxia exposure induces iron overload and ferroptosis in adipose tissue. Antioxidants (Basel) 11 (12), 2367. 10.3390/antiox11122367 36552575 PMC9774922

