# Supplementary material for: Frontiers and hotspots of adipose tissue and NAFLD: a bibliometric analysis from 2002 to 2022
Source: Front Physiol. 2023 Dec 21;14:1278952. doi: 10.3389/fphys.2023.1278952 (PMC10768199; doi:10.3389/fphys.2023.1278952)
Supplement: Supplementary file 1 [file Image1.pdf]

Supplementary Material

Frontiers and hotspots of adipose tissue and NAFLD：A bibliometric analysis from 2002 to 2022

Shuxiao Gu[1+], Yanfang Qiao[1+], Susu Liu[1], Shuangjie Yang[1], Shibo Cong[1], Sili Wang[1], Deshuai Yu[2], Wei Wang[1]* and Xinlou Chai[1]*
[1]School of Traditional Chinese Medicine, Beijing University of Chinese Medicine, Beijing, China
[2]Dongzhimen Hospital, Beijing University of Chinese Medicine, Beijing, China

*** Correspondence:**
Wei Wang: wangwei26960@126.com
Xinlou Chai: mmxin3@126.com

1 Supplementary Figures

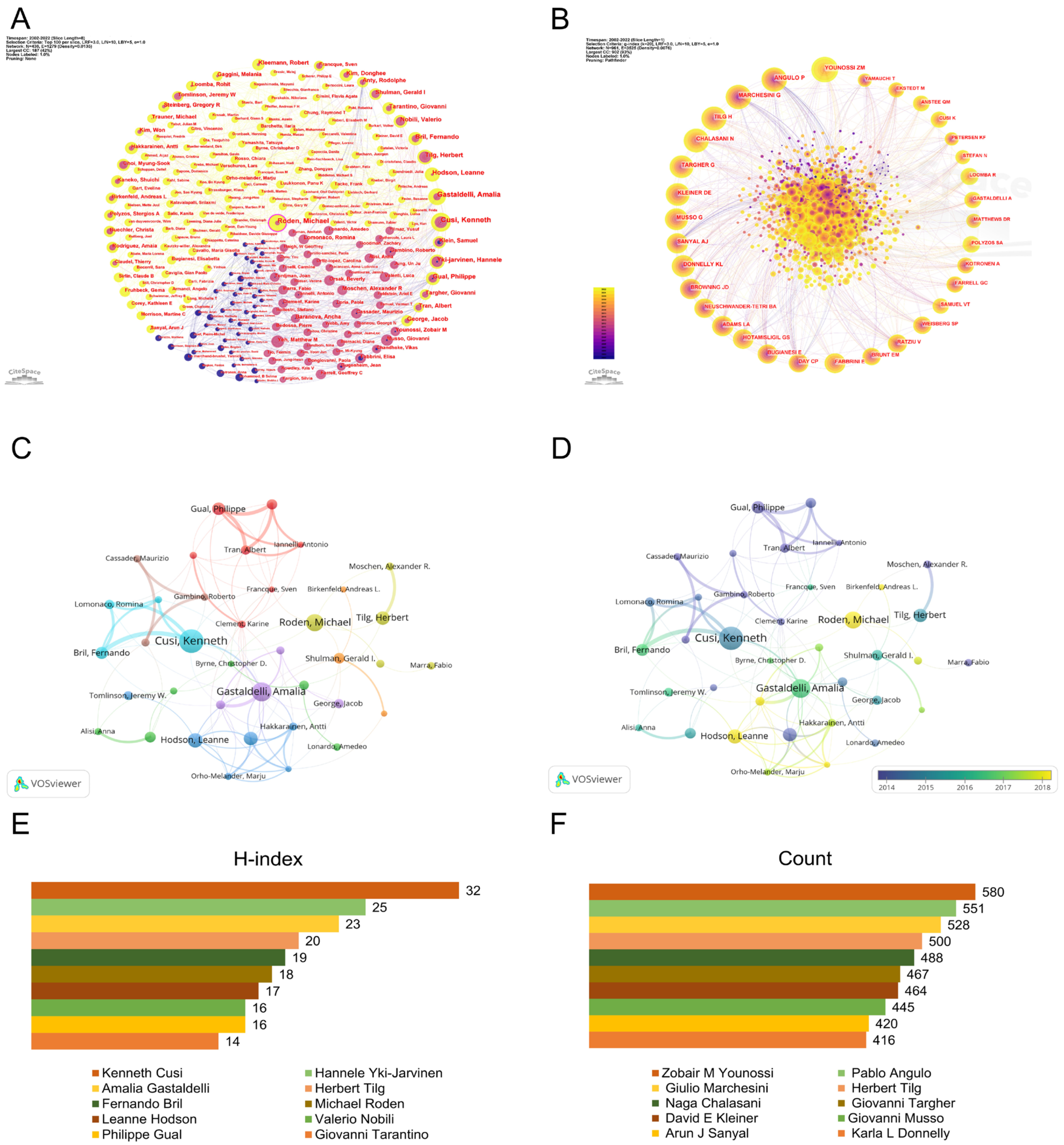

Supplementary Figure 1. Contributions of authors to the research on AT and NAFLD. (A) A visual map among authors based on CiteSpace. (B) A visual map among co-cited authors based on CiteSpace. (C) A visual map among authors based on VOSviewer. (D) Overlay visualization among

authors based on VOSviewer. (E) Top 10 authors in terms of H-index. (F) Top 10 co-cited authors in terms of citations.

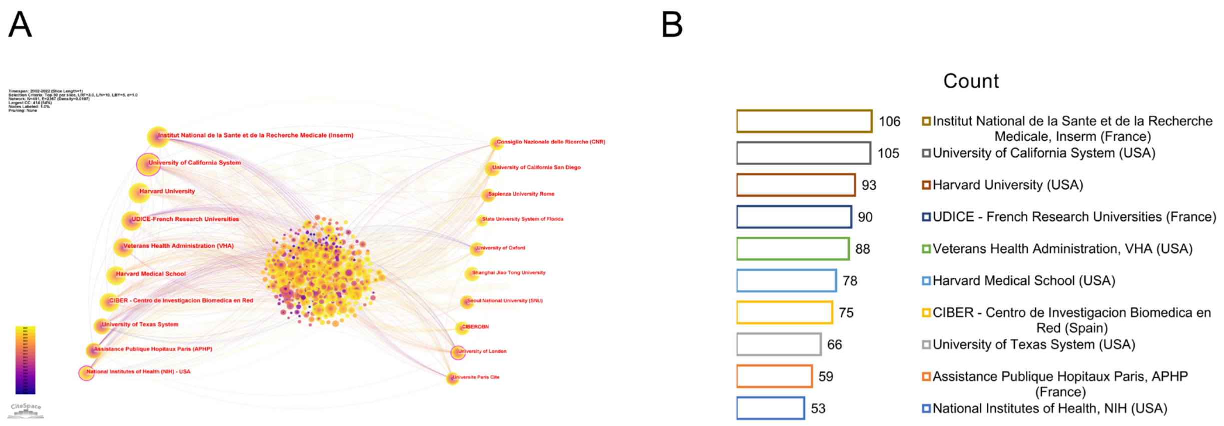

Supplementary Figure 2. Visualization of institutions in AT and NAFLD. (A) A visual map among institutions based on CiteSpace. (B) Top 10 institutions in terms of publications.
